# Sex differences in management and outcomes in pheochromocytomas and paragangliomas

**DOI:** 10.3389/fendo.2025.1597908

**Published:** 2025-06-16

**Authors:** Måns A. J. Dahl, Jan Calissendorff, Henrik Falhammar

**Affiliations:** ^1^ Department of Molecular Medicine and Surgery, Karolinska Institutet, Stockholm, Sweden; ^2^ Department of Endocrinology, Karolinska University Hospital, Stockholm, Sweden

**Keywords:** PPGL, gender, diabetes, hypertension, survival, adrenal medullary tumor

## Abstract

**Purpose:**

The aim of this study was to investigate sex differences in the management and outcomes of patients with pheochromocytomas and paragangliomas (PPGLs).

**Methods:**

This is a retrospective cohort study including all patients diagnosed with PPGLs attending the Department of Endocrinology at Karolinska University Hospital between June 2005 and August 2024. The collected data included patient characteristics, biochemical, genetical, pharmacological and vital parameters noted during initial PPGL presentation and during follow-up, including survival.

**Results:**

In total, 196 patients diagnosed with PPGLs (108 females and 88 males) were included. Paragangliomas were more prevalent in females than in males (23.1% vs 11.4%, P=0.04). Females required a lower final dose of preoperative phenoxybenzamine (50.8 ± 19.8 vs 87.5 ± 75.7 mg, P=0.04), while the final dose of preoperative doxazosin was non-significant lower (22.3 ± 13.6 vs 26.0 ± 13.9 mg, P=0.07). Moreover, females were less likely having laparoscopic surgery than males (55.2% vs 71.1%, P=0.03). After surgery, more females achieved remission from their type 2 diabetes compared to males (23.4% vs 11.8%, P=0.04). Despite similar age at diagnosis and similar follow-up time, no sex differences were identified in metastasis risk, blood pressure outcomes after surgery, or survival.

**Conclusion:**

Females presented more often with paragangliomas which may explain why they were less likely to have laparoscopic surgery. Remission of type 2 diabetes occurred more commonly in females after surgery. Most other outcomes were similar between sexes. More research is needed to explore differences in outcomes between sexes in PPGLs.

## Introduction

Pheochromocytoma and sympathetic paraganglioma (PPGLs) are rare neuroendocrine, catecholamine-secreting tumors. Pheochromocytomas (PCC) derive from chromaffin cells of the adrenal medulla whereas the catecholamine-secreting sympathetic paragangliomas (PGL) arise from extra-adrenal paraganglia tissue (1, 2). PGLs account for 15-20% of PPGLs whereas PCC comprise 80-85% (1).

Symptoms of PPGLs mainly stem from excess catecholamines and may include constant or paroxysmal hypertension, glucose intolerance, pallor, anxiety, and the classic triad, i.e. headaches, diaphoresis and palpitations (3–6). These symptoms can mimic many other diseases, making PPGLs difficult conditions to suspect. To establish a PPGL diagnosis, excess concentrations of plasma free metanephrines or fractionated metanephrines in urine, as well as histopathological proof of a tumor are typically needed, although rarely, the tumor can be silent (1). Early diagnosis and treatment are vital to avoid complications and mortality (7, 8).

Surgery is considered the only curative treatment for PPGLs and preoperative management by a multidisciplinary team is recommended (9, 10). Even if patients are normotensive and/or asymptomatic, an episodic surge in catecholamines can be triggered by surgery, anesthesia, positional changes, or certain medications. These catecholamine surges may cause hypertensive crises, pulmonary edema, arrhythmias, myocardial infarction and multiorgan failure (3, 5, 11). Repeated episodes of catecholamine surges can result in life-threatening catecholaminergic cardiomyopathy, i.e., Takotsubo cardiomyopathy (5, 12). Therefore, pharmacological treatment is important pre-operatively and is achieved with alpha-adrenoreceptor blockers as well as sometimes other additional antihypertensive to reach normotension (9).

PPGLs are mostly benign but all harbor malignant potential (13). The risk of metastases or recurrence is approximately 15% for PCC and 50% for PGL over at least 10 years follow-up (9, 13). Malignant PPGLs are defined by the presence of metastases (9). Patients diagnosed with PPGL should be followed up for at least 10 years to discover recurrences and metastases, and life-long if a genetic cause has been found (9, 14).

Hitherto, sex differences in PPGL are scarcely studied and have only focused on the clinical presentation (4, 15, 16). However, no study has investigated sex differences in management or outcomes. A deeper understanding concerning any underlying sex-related variations could contribute to improved management in the future. Thus, this study aimed to investigate sex differences in management and outcome in patients with PPGLs.

## Methods

This is a retrospective study of patients with PPGLs managed at the Department of Endocrinology, Karolinska University Hospital, Stockholm, Sweden, between June 2005 and August 2024. Patients were included if they had an International Classification of Diseases version 10 (ICD-10) code of E27.5 (Adrenomedullary hyperfunction) and/or C74.1 (Malignant neoplasm: Medulla of adrenal gland). Patients with biochemical and radiological diagnosis were included in the study when histological examination was not available. Each patient was included in the cohort only once, i.e., subsequent recurrences or metastases were only included in the follow-up analysis. Postoperative variables include patients who achieved adequate control with alpha-blockade therapy in lieu of surgery. Preoperative measurements represent values at the time of diagnosis, while postoperative measurements correspond to values measured at the first endocrine follow-up 3–6 months after surgery. Management guidelines for females and males were the same during the study period.

The electronic medical records were manually reviewed for each case. The following parameters were collected for analyses: sex, age at surgery, age at diagnosis, tumor classification, tumor size, presence of metastasis at diagnosis, year of metastasis, type of surgery (laparoscopic surgery/open surgery/converted surgery), surgical complications, length of hospital stay after surgery, follow-up time, recurrence, PPGL metastases and survival. Complications were defined as adverse clinical events occurring intraoperatively or during the postoperative period within the same hospital admission. These included surgical method conversions, massive bleedings, infections, organ injuries, respiratory failures, and cardiovascular events, among others.

The following biochemical, pharmacological and status parameters were collected for analyses: preoperative alpha-adrenoreceptor blockers, duration and final dose of alpha-blocker treatment, genetics, systolic blood pressure (SBP) and diastolic blood pressure (DBP) before and after surgery, number of antihypertensive drugs before and after surgery, Secondary type 2 diabetes mellitus or type 2 diabetes mellitus (Secondary/T2DM) status before and after surgery, antidiabetic medications before and after surgery and glucose abnormalities including prediabetes before and after surgery.

Improved blood pressure was defined as a postoperative reduction of SBP and DBP of at least 10mmHg and/or a reduction in antihypertensive medication. T2DM was defined according to the World Health Organization’s (WHO) criteria: fasting plasma glucose ≥7.0 mmol/L (126 mg/dL) or two-hour plasma glucose ≥11.1 mmol/L (200 mg/dL) or a random venous plasma glucose concentration > 11.1 mmol/L (24). Prediabetes was defined as HbA1c 42–47 mmol/mol and/or random plasma glucose 7.8–11.0 mmol/L and/or fasting plasma glucose 6.0–6.9 mmol/L. For more details on the Methods, please see elsewhere (7, 15, 17).

### Statistical analysis

The cohort was divided into females and males, and all data were analyzed to assess differences between sexes. Continuous data were presented as mean ± standard deviation (SD) if normally distributed, otherwise as median and range. Shapiro – Wilks test was used to assess normal distribution. For continuous variables, unpaired t-tests were applied when normality was met, and the Mann-Whitney U test was used otherwise. Categorical data were presented as a count (n) and percentage (%). If the analysis included missing values, the total sample size and corresponding percentages were reported. Comparisons of categorical variables were performed using Fisher’s exact test. Kaplan-Meier estimates were employed for time-to-event analyses.

Statistical significance was defined as P < 0.05. All statistical analyzes were conducted using R, version 4.4.2 (Vienna, Austria. URL: https://www.R-project.org/).

## Results

In total, 196 patients were included in the analysis, consisting of 108 (55.1%) females and 88 (44.9%) males. Patient characteristics, including preoperative cardiovascular events, and management variables before surgery are presented in **Table 1**. Females were more likely to present with PGL compared to males (23.1% vs 11.4%, P = 0.04). Females and males were diagnosed at a similar age (53.8 ± 18.5 vs 56.2 ± 15.4 years, P = 0.45). Preoperative cardiovascular events - including Takotsubo syndrome, ischemic heart disease, stroke, and arrhythmia - occurred at similar rates in both female and male patients (32.1% vs 26.4%, P = 0.43).

**Table 1:** Baseline characteristics in patients with pheochromocytoma or sympathetic paraganglioma.

	Total (n = 196)	Females (n = 108)	Males (n = 88)	*P* value
**Pheochromocytoma (n)**	161 (82.1%)	83 (76.9%)	78 (88.6%)	**0.04**
**Paraganglioma (n)**	35 (17.9%)	25 (23.1%)	10 (11.4%)	**0.04**
**Mean age at dx (yrs)**	54.9 ± 17.2	53.8 ± 18.5	56.2 ± 15.4	0.45
**CV event preop (n)**	57/193 (29.5%)	34/106 (32.1%)	23/87 (26.4%)	0.43
**Tumor size (mm)**	48.63 ± 27.45	49.09 ± 27.60	48.07 ± 27.41	0.50
Positive gene testing*	32/123 (26.0%)	20/71 (28.2%)	12/52 (23.1%)	0.68
**Received alpha blockage**	184 (93.9%)	102 (94.4%)	82 (93.2%)	0.77
**Received doxazosin**	158 (80.6%)	89 (82.4%)	69 (78.4%)	0.59
**Received phenoxybenzamine**	25 (12.8%)	13 (12.0%)	12 (13.6%)	0.83
**Days on alpha blockage prior to sx**	51.5 [0-360]	46.5 [0-360]	60 [0-330]	0.89
**Final doxazosin dose preop (mg)**	23.9 ± 13.8	22.3 ± 13.6	26.0 ± 13.9	*0.07*
**Final phenoxybenzamine dose preop (mg)**	69.2 ± 57.2	50.8 ± 19.8	87.5 ± 75.7	**0.04**
Surgery**	188 (95.9%)	105 (97.2%)	83 (94.3%)	0.47
**Laparoscopic sx**	117/188 (62.2%)	58/105 (55.2%)	59/83 (71.1%)	**0.03**
**Open sx**	59/188 (31.4%)	39/105 (37.1%)	20/83 (24.1%)	*0.06*
**Converted to open sx**	12/188 (6.4%)	8/105 (7.6%)	4/83 (4.8%)	0.55
**LOS postop (days)**	4 [2-75]	4 [2-44]	5 [2-75]	0.24
Complications*** (n)	39/188 (20.7%)	18/105 (17.1%)	21/83 (25.3%)	0.21
**Follow-up time (yrs)**	10.96 ± 8.75	11.19 ± 9.10	10.67 ± 8.34	0.75
**Deceased (n)**	40 (20.4%)	17 (15.7%)	23 (26.1%)	*0.08*
**Deaths caused by PPGL (n)**	7/36 (19.4%)	4/16 (25.0%)	3/20 (15.0%)	0.68
**Metastatic PPGL developing during follow-up (n)**	20 (10.2%)	11 (10.2%)	9 (10.2%)	1
**Mean year of metastases appearance**	4.99 ± 3.63	4.26 ± 3.45	5.91 ± 3.86	0.36

Results are presented as numbers (n), percentages (%), means and standard deviations (SD) or medians and min-max values [range]. Bold, P < 0.05. Italic, P = 0.05–0.09. P value evaluates the difference between the females and males. *Females tested positive for NF1 (n = 5), RET (n = 8), SDHB (n = 5), VHL (n = 2). Males tested positive for NF1 (n = 5), RET (n = 5), SDHA (n=1), SDHB (n = 1). **Eight patients were ineligible for surgery, two refused surgery, two had extensive PPGL metastases, one presented with widespread adenocarcinoma, one was treated palliatively for multimorbidity and two died before surgery. ***Complications include all adverse clinical events occurring intraoperatively or during the postoperative period within the same hospital admission. dx diagnosis, yrs years, sx surgery, CV cardiovascular, LOS postop number of days admitted in hospital after surgery.

Comparing patient characteristics and surgical management, categorized by sex.

### Surgery

Parameters related to surgery and preoperative treatment are shown in **Table 1**. Prior to surgery, the majority of patients underwent initial treatment with alpha-blockers, mostly doxazosin or phenoxybenzamine, and one with phentolamine. Of the remaining cases not receiving alpha-blockers, four were discovered during surgery for suspected alternative tumors, two had died before treatment initiation, one was managed palliatively with no PPGL symptoms, and the remaining five were not treated due to mild biochemical and asymptomatic disease or patient refusal. The median duration of preoperative alpha-blocker therapy was 46.5 days (range: 0–360) in females and 60 days (range: 0–330) in males (P = 0.89). Alpha-blockers were used in 94.4% of females and 93.2% of males (P = 0.77). The majority of these patients were treated with doxazosin (82.4% of females vs 78.4% of males, P = 0.59). Patients who were treated more than two decades ago primarily received preoperative therapy with phenoxybenzamine (12.0% of females vs 13.6% of males P = 0.83). The mean dose of doxazosin was 22.3 ± 13.6 mg for females and 26.0 ± 13.9 mg for males (P = 0.07). In the group of phenoxybenzamine, the mean dose was 50.8 ± 19.8 mg for females and 87.5 ± 75.7 mg for males (P = 0.04). Eight patients (4.1%) did not undergo surgery due to the following reasons: two patients refused surgery, two had PPGL with extensive metastases, one presented with widespread adenocarcinoma, one was treated palliatively for multiple morbidities and two died before surgery. Where feasible, these patients were managed with alpha-blockers. Among those not having surgery, three (2.8%) were female, and five (5.7%) were male (P = 0.47).

Females underwent laparoscopic surgery less frequently than males (55.2% vs 71.1%, P = 0.03). In instances where laparoscopic surgery was initiated but could not be completed due to encountered intraoperative challenges, the operating surgeon could opt for conversion to open surgery. Conversions were required in eight females (7.6%) and four males (4.8%) (P = 0.55). Open surgery was performed in cases where other minimally invasive surgical approaches were deemed not to be suitable. A total of 39 (37.1%) females and 20 (24.1%) males underwent open surgery (P = 0.06). Tumor sizes were similar between females and males (49.1 ± 27.6 vs 48.1 ± 27.4 mm, P = 0.50).

The data regarding complications during surgery showed no difference between sexes (17.1% vs 25.3%, P = 0.21). Likewise, length of stay after surgery (LOS) showed no difference between females and males (4 [2-44] vs 5 [2-75] days, P = 0.24).

### Comparison of glycemic disturbances between sexes

Associations between sex and glycemic disturbances are presented in **Table 2**. At diagnosis, Secondary/T2DM was present in a similar proportion in females and males (28.7% vs 23.9%, P = 0.52). When all glucose abnormalities – including patients with prediabetes, Secondary/T2DM and one patient with type 1 diabetes mellitus– were assessed, prevalence between females and males was also similar (38.9% vs 37.5%, P = 1).

**Table 2:** Glycemic abnormalities in patients with pheochromocytoma or sympathetic paraganglioma.

	Total (n = 196)	Females (n = 108)	Males (n = 88)	*P* value
**Secondary/T2DM preop (n)**	52 (26.5%)	31 (28.7%)	21 (23.9%)	0.52
Any glycemic disturbance* preop (n)	75 (38.3%)	42 (38.9%)	33 (37.5%)	1
**T2DM postop (n)**	17/193 (8.8%)	6/107 (5.6%)	11/85 (12.9)	0.12
**Secondary/T2DM remission (n)**	35/192 (18.8%)	25/107 (23.4%)	10/85 (11.8%)	**0.04**
Glucose improvement** (n)	42/192 (21.9%)	26/107 (24.3%)	16/85 (18.8%)	0.39

Results are presented as numbers (n) and percentages (%) Bold, P < 0.05. P value evaluates the difference between the females and males. Postop parameters were assessed at the first endocrine follow-up outpatient visit after surgery, 3–6 months after surgery. *Any glycemic disturbance includes patients with diabetes mellitus type 1 and 2 as well as prediabetic patients (prediabetes is defined by HbA1c levels between 42–47 mmol/mol or fasting plasma glucose concentrations of 6.0–6.9 mmol/L or random plasma glucose levels ranging between 7.8–11.0 mmol/L). **Glucose improvement was defined as either T2DM remission or a reduction in dose of antidiabetic drugs. Abbreviations: Secondary/T2DM Secondary cause of type 2 diabetes mellitus or type 2 diabetes mellitus.

Effects of treatment on glycemic status, categorized by sex.

Postoperatively, females demonstrated a non-significant lower prevalence of Secondary/T2DM than males (5.6% vs 12.9%, P = 0.12). Females achieved Secondary/T2DM remission at a higher rate than males (23.4% vs 11.8%, P = 0.04). Measuring overall glucose improvement, females and males were quite similar (24.3% vs 18.8%, P = 0.39).

### Comparison of blood pressure parameters between sexes

Blood pressure parameters are presented in **Table 3**. Preoperative systolic and diastolic blood pressure (SBP and DBP) was similar between sexes (**Table 3**). Postoperative SBP had a tendency to be lower in females than in males (122.8 ± 15.3 vs 126.3 ± 14.1, P = 0.06). Postoperative DBP showed similar levels between sexes. SBP and DBP decrease after surgery were similar between sexes.

**Table 3:** Blood pressure in patients with pheochromocytoma or sympathetic paraganglioma.

	Total (n = 196)	Females (n = 108)	Males (n = 88)	*P* value
**Systolic BP preop (mmHg)**	151.5 ± 31.0	150.66 ± 31.0	152.4 ± 31.2	0.77
**Diastolic BP preop (mmHg)**	87.7 ± 17.2	88.1 ± 17.9	87.1 ± 16.4	0.82
**Systolic BP postop (mmHg)**	124.3 ± 14.8	122.8 ± 15.3	126.3 ± 14.1	*0.06*
**Diastolic BP postop (mmHg)**	75.7 ± 9.8	75.0 ± 9.8	76.5 ± 9.7	0.35
Δ Systolic BP* (mmHg)	28.1 ± 30.7	29.2 ± 30.7	26.6 ± 30.9	0.52
Δ Diastolic BP** (mmHg)	12.8 ± 19.2	14.3 ± 20.8	11.0 ± 17.0	0.46
Improved BP after surgery*** (n)	161/191 (84.3%)	88/105 (83.8%)	73/86 (84.9%)	1
**BP medication preop (n)**	1.69 ± 1.06	1.66 ± 1.06	1.73 ± 1.07	0.86
**BP medication postop (n)**	0.54 ± 0.89	0.52 ± 0.83	0.57 = ± 0.96	0.97
**Mean reduction of BP medication (n)**	1.18	1.16	1.21	0.86

Results are presented as numbers (n), percentages (%), means and standard deviations (SD) Bold, P < 0.05. Italic, P = 0.05–0.09. P value evaluates the difference between the females and males. Postoperative parameters were assessed at the first endocrine follow-up outpatient visit after surgery. The postop cohort includes eight patients who achieved adequate control with alpha blockage. *Δ Systolic BP, the difference in the change in mean systolic BP from preoperative to postoperative measurements. **Δ Diastolic BP, the difference in the change in mean diastolic BP from preoperative to postoperative measurements. ***Improvement BP defined as reduction of systolic and diastolic BP of at least 10 mmHg and/or reduction in BP medications. Abbreviations: BP blood pressure.

Effects of surgery on blood pressure in patients with pheochromocytoma or sympathetic paraganglioma, categorized by sex.

Improved blood pressure, defined as a postoperative reduction of SBP and DBP of at least 10mmHg and/or a reduction in antihypertensive medication, was comparable between females and males (83.8% vs 84.9%, P = 1). Regarding antihypertensive medication use, females and males exhibited similar results, preoperatively (1.66 ± 1.06 vs 1.73 ± 1.07, *P* = 0.86) and postoperatively (0.52 ± 0.83 vs 0.57 ± 0.96, P = 0.97). The mean reduction of antihypertensive medications was similar between sexes (1.16 for females vs 1.21 for males, P = 0.86).

### Comparison of metastases and mortality between sexes

**Figure 1:**
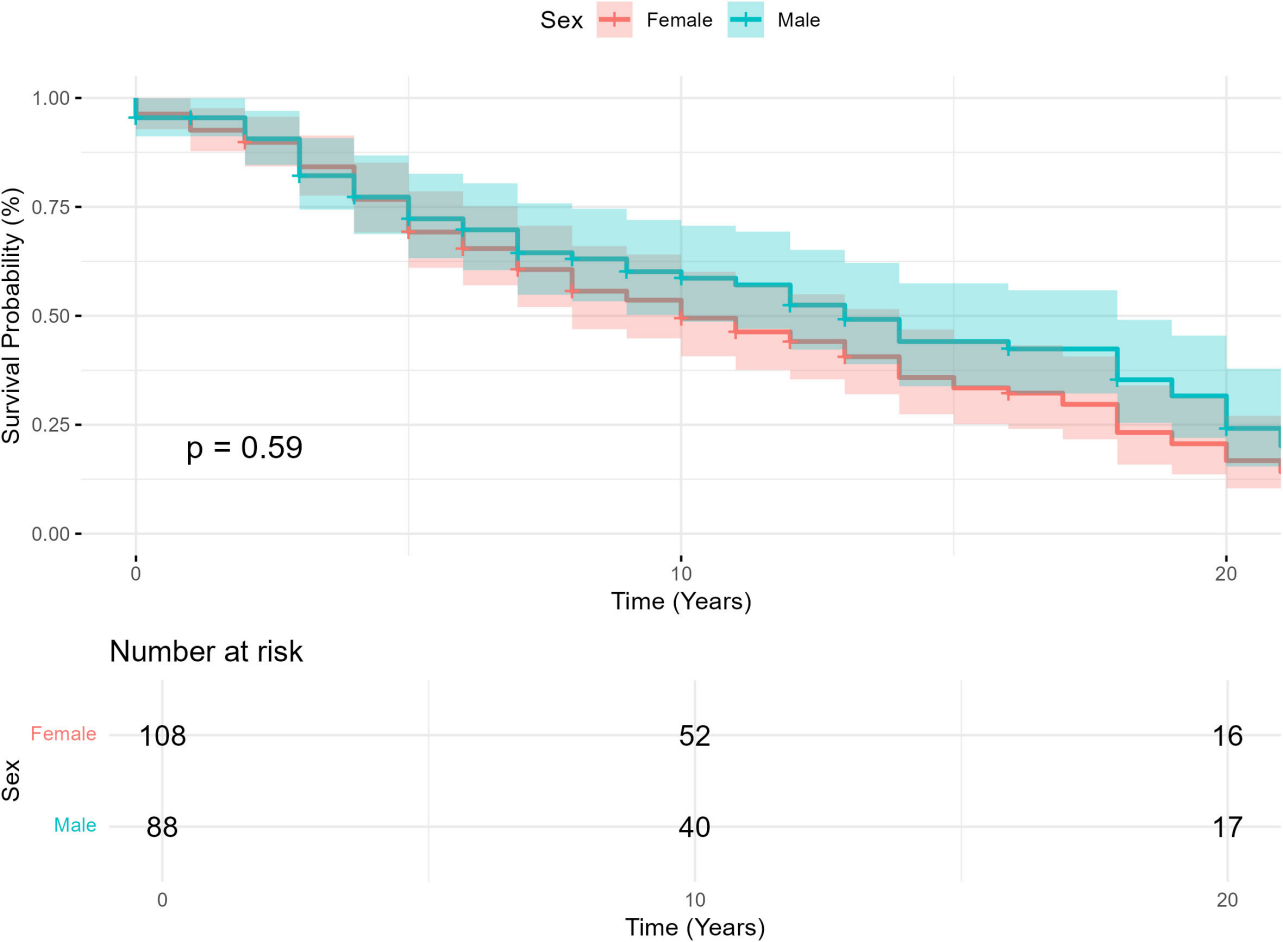
Kaplan-Meier survival curve illustrating survival probability in patients diagnosed with pheochromocytoma or sympathetic paraganglioma, categorized by sex.

Follow-up time was similar between females and males (11.2 ± 9.1 vs 10.7 ± 8.3 years, P = 0.75). There was no difference in the number of patients developing PPGL metastases in females (10.2%) and in males (10.2%) (P = 1). Also, metastases developed at approximately the same time following surgery (4.26 ± 3.45 in females vs 5.91 ± 3.86 years in males, P = 0.36). In cases of metastasis, patients were primarily treated with alpha-blockers, surgery and/or peptide receptor radionuclide therapy (PRRT) with either lutetium or meta-iodobenzylguanidine (MIBG). In total, 20.4% of patients died (15.7% of females vs 26.1% of males, P = 0.08). Two male patients died prior to surgery due to PCC complications and to cardiomyopathy secondary to myeloma respectively. The remaining deaths occurred years later. Among all reported deaths, the cause of death was identified in 36 cases. Deaths attributed to by PPGLs were similar between sexes (25.0% of female deaths and 15.0% of male deaths, P = 0.68) (**Table 1**). A Kaplan-Meier survival analysis was used to evaluate survival following diagnosis between sexes (**Figure 1**). The survival curve demonstrated no statistically significant difference between sexes (P = 0.59).

## Discussion

For the first time, a cohort specifically aimed to investigate differences in management and outcome between females and males with PPGL was studied. Females were more likely to present with PGL compared to males, which may explain the lower laparoscopic rate in females. Females with PPGL demonstrated a more favorable post-surgical remission rate of T2DM.

There were few significant differences in group characteristics between sexes that could substantially impact the reliability of outcome variables. The cohort showed a greater prevalence of PPGL in females than males, a sex distribution supported by others (4, 11, 16, 18, 19). Furthermore, PGL was more common in females than in males (23.1% vs 11.4%) in our study, supported by La Salle et al. reporting a slightly higher incidence of PGL among women, despite an overall predominance of PPGL in males (4). This disparity can likely explain why females were less likely to undergo a laparoscopic procedure, since PGL usually require open surgery due to their often challenging localization (1, 9).

### Alpha-blockage management

The final dose of phenoxybenzamine was higher in males than in females. In addition, males tended to need higher final doses of doxazosin. It can be viewed as a limitation that not all patients were treated with the same alpha-blockage. If all patients with phenoxybenzamine had been treated with doxazosin, the current preferred choice of alpha-blocker due to fewer side-effects (1), a significant difference in dose between female and male patients might have been observed. While no study have discussed potential differences in dose of alpha-blockage between sexes, females have been found to be more sensitive to alpha-adrenergic receptor stimulation (20). Moreover, females using anti-hypertensive medications achieved greater blood pressure control than males at the same dose, potentially due to natural differences in pharmacokinetics and pharmacodynamics (21). We hypothesize that this applies to alpha-blockers as well.

### Glycemic abnormalities

Our study found that a quarter of patients had preoperative Secondary/T2DM, with no significant sex-specific difference, which is also observed in most other studies (22, 23), but not all (4). However, there is a great variation in Secondary/T2DM prevalence as some studies have reported preoperative Secondary/T2DM to be existent in up to 50% of PPGLs cases (24). Interestingly, although we could not find any sex difference in the overall glucose improvement, females displayed a significantly higher rate of Secondary/T2DM remission after surgery. Studies have shown higher preoperative metanephrine concentrations and older age to be risk factors for persistent diabetes after surgery (25–27). Furthermore, lower body mass index and a duration of disease shorter than 3 years were predictive factors for Secondary/T2DM remission (27). To our knowledge, none has shown sex being a factor.

Our findings are interesting when considering potential complications after surgery. Araki et al. showed that 21% out of 49 patients undergoing surgery for PPGL, experienced severe post-excisional hypoglycemia (28). This considered, we can speculate that females may be more likely to suffer severe hypoglycemia after tumor removal, due to the recovery of insulin secretion after tumor surgery (23). However, our data did not support this, as we observed similar complication risk after surgery between sexes, also supported by others (29). Nevertheless, clinicians need to be aware of hypoglycemia as a complication after surgery, maybe particularly among female patients.

### Blood pressure

PPGL is considered a potentially curable cause of secondary hypertension (7). We observed a dramatic decrease in blood pressure in both sexes. No significant difference was observed between females and males in any blood pressure measurements, which is in accordance with others (30).

### Risk of recurrence and metastases

No sex difference was found in the long-term risk of developing PPGL recurrence or metastases with 10.2% of both females and males developing PPGL metastases with time. This finding is consistent with the estimated overall metastasis risk shown in previous research stating that approximately 10-20% of all patients with PPGLs develop metastases (31). Genetic predisposition is a well-established predictor of PPGL recurrence (32); however, the prevalence of positive genetic panels and the mean follow-up time were comparable between sexes in our cohort, thereby effectively ruling out genetic predisposition and follow-up time as potential confounders influencing risk of metastasis (32). Hamidi et al. also found similar risk of metastasis between sexes (33). Furthermore, a multicenter observational study conducted by Bechmann et al. showed that 6.7% of patients with PCC had developed distant metastasis with no sex difference (34). A review article comparing different cohorts also described absence of sex-related differences in long-term outcome of PPGLs, even when considering primary malignant or recurrent PCCs (35). In contrast, Plouin et al., comparing 129 patients, showed that metastatic PPGLs were more prevalent in males preoperatively, however, the risk of subsequent recurrence was not found to be associated with sex (30).

PGLs have been found to have more than double the metastatic potential compared to PCCs (36). Thus, having more females with PGLs in our study, one might have expected a higher incidence of metastases amongst females. This may suggest that other factors beyond tumor type and genetic variants influence metastatic potential. Future research aimed at assessing metastatic potential individually between PCC and PGL comparing sex may be relevant.

### Survival

Mortality was found to be higher than that reported in some long-term follow-up studies (37, 38). This may be explained by the higher mean age at diagnosis, larger tumor size, as well as a higher frequency of metastasis in our study than those compared. Survival among patients with PPGL was similar between the sexes. In our study, age at diagnosis and follow-up time were similar between sexes with some being followed for up to 20 years. The similar survival is particularly interesting, considering males have a shorter average lifespan (39). Therefore, it would be possible to expect that males with PPGL would have a shorter survival compared to female patients. In contrast, our findings may suggest a potential for higher mortality in females with PPGL. This observation possibly reflects females being more likely to have PGLs in our study, which have been shown to harbor a more malignant potential and be more difficult to surgically excise (9). However, a retrospective single center study by Raber et al., found that females with PPGLs had longer survival (40). Moreover, Plouin et al. demonstrated that sex was not associated to the PCC-free survival (30).

### Strengths and limitations

This study has several strengths with the most notable one being that it is the first study to compare sexes in management and outcome in patients with PPGL. With PPGLs being particularly rare diseases, gathering a cohort of this size from a single center is difficult. However, the main limitation lies in its retrospective design, introducing potential biases, including selection bias due to the exclusion of patients with substantial missing data. Retrospective studies are also prone to confounders that can only be acknowledged and addressed in the discussion. Although this cohort is large relative to the topic, it may still have power issues in some analyses. Furthermore, having the strength of being the first study to address the topic of sex differences in management and outcomes also limits the critical depth since there will be absence of comparable studies. Another shortcoming of this study, related to it being retrospective, was that a small proportion of patients had missing data in certain parameters which were impossible to collect in hindsight.

Moreover, with this study being single center, we reduced confounders such as variability in clinical protocol, enhancing the reliability of all patients having similar prerequisites for management and treatment. Conversely, this acts as a limitation since we are unable to prove that it is applicable to a broader population. Finally, many of our patients with PPGLs missed genetic results. This is partially explained by a stricter indication to evaluate the genetics in PPGLs previously but also partially explained by the fact that many of our patients had been included in a study of genetics in PPGLs and the samples had not been analyzed at the time of the analysis of this cohort.

## Conclusion

This is the first study to comprehensively investigate sex difference in management and outcomes in patients with PPGL. Most outcomes were similar between sexes. Females were diagnosed with PGLs more frequently and were less commonly treated with laparoscopic surgery. Regarding preoperative management, males required higher final doses of alpha-blocker prior to surgery. Moreover, females were more likely to achieve remission of T2DM following surgery compared to males. Survival, recurrence and metastasis rates were similar between sexes during follow-up.

## Data Availability

The datasets presented in this article are not readily available because privacy and legal reason. Requests to access the datasets should be directed to Henrik Falhammar.
